# Pathology of Intermittent Diseases

**Published:** 1848

**Authors:** 


### ARTICLE XIII.

### *Pathology of Intermittent Diseases.*

Dr. Amand Beaupoil has just published in the *Gazette Medicale*, an extremely interesting paper, wherein is treated the disputed question of the localization of intermittent fever. It would give us pleasure to analyze this dissertation fully, for it is worthy of notice, but we are obliged, for want of space, to confine ourselves to the conclusions: 1. The seat of the intermittent phenomena cannot be placed in any organ or system of organs to the exclusion of others. 2. The intermittent may become connected with any other affection, and form with it a sort of unity or special morbid entity; the disulphate of quinine is a specific for this compound intermittence, as well as for simple ague. 3. Intermittence is often combined with an organic affection, in the manner of complication; it behooves us, then, to administer the specific remedy simultaneously with the means indicated by the organic lesion. The treatment ought, in fact, to be double. 4. When the intermittence is independent of any organo-pathological rhythm of organic contraction and expansion, and this state produces an alteration in the amount and variations of animal heat, and constitutes intermittent fever. 5. It will, then, be seen that ague may be simple, compound, or complicated. 6. The intermittent phenomena may be attributed to the effect of miasmata on the economy; the noxious emanations being modified by periodic alternations of heat and cold, of dryness and humidity, of light and darkness, of sleep and waking, and of all influences which act in the same way. 7. The nervous element evidently plays an important part in the production of intermittence, brought on by the causes just mentioned; it seems, indeed, that the influence of this nervons element is altegether indispensable. 8. Enlargement of the spleen is the *effect*, and not the *cause*, of intermittent fever; it is an indication for large doses of quinine.—*Lancet.*

